# NOTCH and DNA repair pathways are more frequently targeted by genomic alterations in inflammatory than in non‐inflammatory breast cancers

**DOI:** 10.1002/1878-0261.12621

**Published:** 2020-02-05

**Authors:** François Bertucci, Charlotte Rypens, Pascal Finetti, Arnaud Guille, José Adélaïde, Audrey Monneur, Nadine Carbuccia, Séverine Garnier, Piet Dirix, Anthony Gonçalves, Peter Vermeulen, Bisrat G. Debeb, Xiaoping Wang, Luc Dirix, Naoto T. Ueno, Patrice Viens, Massimo Cristofanilli, Max Chaffanet, Daniel Birnbaum, Steven Van Laere

**Affiliations:** ^1^ Laboratoire d'Oncologie Prédictive Centre de Recherche en Cancérologie de Marseille (CRCM) Inserm U1068 CNRS UMR7258 Institut Paoli‐Calmettes Aix‐Marseille Université France; ^2^ Département d'Oncologie Médicale Institut Paoli‐Calmettes Marseille France; ^3^ Translational Cancer Research Unit and Center for Oncological Research (CORE) Faculty of Medicine and Health Sciences GZA Hospitals Sint‐Augustinus and University of Antwerp Wilrijk Antwerp Belgium; ^4^ MD Anderson Morgan Welch Inflammatory Breast Cancer Research Program and Clinic The University of Texas MD Anderson Cancer Center Houston TX USA; ^5^ Division of Hematology and Oncology Robert H Lurie Comprehensive Cancer Center Northwestern University Chicago IL USA

**Keywords:** copy number profiling, DNA repair, inflammatory breast cancer, NOTCH, sequencing, targeted therapy

## Abstract

Inflammatory breast cancer (IBC) is the most pro‐metastatic form of breast cancer. Better understanding of its pathophysiology and identification of actionable genetic alterations (AGAs) are crucial to improve systemic treatment. We aimed to define the DNA profiles of IBC *vs* noninflammatory breast cancer (non‐IBC) clinical samples in terms of copy number alterations (CNAs), mutations, and AGAs. We applied targeted next‐generation sequencing (tNGS) and array‐comparative genomic hybridization (aCGH) to 57 IBC and 50 non‐IBC samples and pooled these data with four public datasets profiled using NGS and aCGH, leading to a total of 101 IBC and 2351 non‐IBC untreated primary tumors. The respective percentages of each molecular subtype [hormone receptor‐positive (HR+)/HER2−, HER2+, and triple‐negative] were 68%, 15%, and 17% in non‐IBC *vs* 25%, 35%, and 40% in IBC. The comparisons were adjusted for both the molecular subtypes and the American Joint Committee on Cancer (AJCC) stage. The 10 most frequently altered genes in IBCs were *TP53* (63%), *HER2/ERBB2* (30%), *MYC* (27%), *PIK3CA* (21%), *BRCA2* (14%), *CCND1* (13%), *GATA3* (13%), *NOTCH1* (12%), *FGFR1* (11%), and *ARID1A* (10%). The tumor mutational burden was higher in IBC than in non‐IBC. We identified 96 genes with an alteration frequency (*p* < 5% and *q* < 20%) different between IBC and non‐IBC, independently from the molecular subtypes and AJCC stage; 95 were more frequently altered in IBC, including *TP53*, genes involved in the DNA repair (*BRCA2*) and NOTCH pathways, and one (*PIK3CA*) was more frequently altered in non‐IBC. Ninety‐seven percent of IBCs displayed at least one AGA. This percentage was higher than in non‐IBC (87%), notably for drugs targeting DNA repair, NOTCH signaling, and CDK4/6, whose pathways were more frequently altered (DNA repair) or activated (NOTCH and CDK4/6) in IBC than in non‐IBC. The genomic landscape of IBC is different from that of non‐IBC. Enriched AGAs in IBC may explain its aggressiveness and provide clinically relevant targets.

## Graphical abstract

We compared the DNA mutational and copy number profiles and mRNA expression profiles of a large series of IBC and noninflammatory breast cancer (non‐IBC) untreated primary tumors. Compared to non‐IBC samples, IBC samples displayed more frequent alterations in the NOTCH and DNA repair pathways independently form the unbalance in term of molecular subtypes and American Joint Committee on Cancer stages.

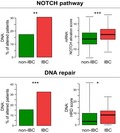

**Abbreviations**
aCGH: array‐comparative genomic hybridization
AGA: actionable genetic alteration
AJCC: American Joint Committee on Cancer
CIViC: Clinical‐Interpretation‐of‐Variants‐in‐Cancer
CNA: copy number alteration
GDKD: Gene‐Drug‐Knowledge‐database
HR+: hormone receptor‐positive
HRD: homologous recombination deficiency
IBC: inflammatory breast cancer
IHC: immunohistochemistry
Mb: megabase
Metabric: Molecular Taxonomy of Breast Cancer International Consortium
NGS: next‐generation sequencing
non‐IBC: noninflammatory breast cancer
SNP: single nucleotide polymorphisms
SNV: single nucleotide variant
TARGET: Tumor‐Alterations‐Relevant‐for‐Genomics‐driven‐Therapy
TCGA: The Cancer Genome Atlas
TMB: tumor mutational burden
TN: triple‐negative
tNGS: targeted next‐generation sequencing
WES: whole‐exome sequencing
WGS: whole‐genome sequencing

## 1 Introduction

Inflammatory breast cancer (IBC) is the most aggressive clinical form of breast cancer (Dawood *et al.*, 2011). Despite therapeutic progresses, ~ 50% of patients die from metastatic relapse. The distinct clinical presentation and aggressive behavior have not translated in design of differential treatment that remains similar to that of stage 3 noninflammatory breast cancer (non‐IBC). Identification of new therapeutic targets and better understanding of the pathophysiology are crucial (Charafe‐Jauffret *et al.*, 2008). Because of the scarcity of disease, ‘omics’ studies remain rare in IBC (Bertucci *et al.*, 2014a). The largest series reported to date is the one that we had collected within the International IBC Consortium (Bertucci *et al.*, 2014b; Masuda *et al.*, 2013; Van Laere *et al.*, 2013), in which we notably showed the overrepresentation of aggressive molecular subtypes (basal, HER2‐enriched, luminal B) when compared with non‐IBC, justifying the need to stratify the IBC/non‐IBC comparison upon the molecular subtypes (Van Laere *et al.*, 2013).

During the last decade, next‐generation sequencing (NGS) led to identification of driver alterations in non‐IBC (Banerji *et al.*, 2012; Ellis *et al.*, 2012; Ferrari *et al.*, 2016; Nik‐Zainal *et al.*, 2012a; Nik‐Zainal *et al.*, 2016; Nik‐Zainal *et al.*, 2012b; Shah *et al.*, 2012; Stephens *et al.*, 2012; The Cancer Genome Atlas, 2012). Precision medicine trials have shown the potential of DNA‐based genomics screening to identify clinically actionable genetic alterations (AGAs) for guiding treatment (Andre *et al.*, 2014; Le Tourneau *et al.*, 2015). Regarding IBC, five NGS‐based studies have been published since 2015 (Goh *et al.*, 2016; Hamm *et al.*, 2016; Liang *et al.*, 2018; Matsuda *et al.*, 2017; Ross *et al.*, 2015). Except the most recent contribution (Liang *et al.*, 2018), they concerned small series ranging from 19 to 53 IBCs, including both untreated primary tumors (between 16 and 25 cases only) and pretreated relapses. The number of tested genes varied between 50 and 255 for the studies using targeted NGS (Hamm *et al.*, 2016; Liang *et al.*, 2018; Matsuda *et al.*, 2017; Ross *et al.*, 2015) and whole‐exome sequencing (WES) (Goh *et al.*, 2016). Few studies directly compared the genomic portraits of primary IBC and non‐IBC, and comparison was never stratified upon the molecular subtypes. However, three of the most recurrently mutated genes (*TP53*, *PIK3CA*, and *HER2*) have clear ties with molecular subtypes [i.e., triple‐negative (TN), luminal, and HER2‐enriched respectively]. The main finding of these studies was an increased tumor mutational burden (TMB) in IBC that translated in the presence of many AGAs with low frequency, but without identification of IBC‐specific driver genes.

Here, we present a large comparative study of untreated primary tumors of IBC and non‐IBC based on NGS data from Institut Paoli‐Calmettes (IPC; Marseille, France) and TCRU (Antwerp, Belgium), pooled with publicly available data (Hamm *et al.*, 2016; Pereira *et al.*, 2016; Ross *et al.*, 2015; The Cancer Genome Atlas, 2012). After adjustment upon both the molecular subtypes and American Joint Committee on Cancer (AJCC) stage, we compared the genomic profiles of IBC and non‐IBC by in terms of DNA mutations and copy number alterations (CNA), TMB, and presence of AGAs.

## 2 Materials and methods

### 2.1 Patients and samples selection

All clinical samples were pretreatment diagnostic samples of primary breast cancers. IBC was clinically defined as T4d according to the international consensus criteria (Dawood *et al.*, 2011), and the samples were diagnostic biopsies (AJCC stages 3–4). Non‐IBC samples were surgical specimens in case of early‐stage disease (stages 1–2) and diagnostic biopsies in case of advanced stage disease (locally advanced: stage 3, and metastatic: stage 4). The whole series included 101 IBCs and 2351 non‐IBCs, collected from six different sources (Table S1).

Forty‐four IBC and 50 non‐IBC samples were from patients consecutively treated at IPC, and 13 IBC samples were from patients consecutively treated at the General Hospital Sint‐Augustinus (TCRU). Extraction of tumor DNA, quality control, and concentration assessment were done as described (Bertucci *et al.*, 2016). Each patient gave written informed consent, and the study was approved by the respective institutional review boards. The study methodology conformed to the standards set by the Declaration of Helsinki. The selection criteria included available frozen sample, tumor cellularity assessment to guide DNA extraction (> 50%), good‐quality extracted tumor DNA, and available clinicopathological data. These samples were pooled with four public series of similarly defined IBC and non‐IBC samples profiled by NGS [and array‐comparative genomic hybridization (aCGH) for two series]. The Ross’ (Ross *et al.*, 2015) and Hamm's (Hamm *et al.*, 2016) series included 25 and 17 IBC samples, respectively; the TCGA series (Cancer Genome Atlas, 2012) included two IBC and 988 non‐IBC samples; the Molecular Taxonomy of Breast Cancer International Consortium (Metabric) series included 1313 non‐IBC samples (Pereira *et al.*, 2016). The molecular subtype of tumors based upon immunohistochemistry (IHC) was defined as HR+/HER2− when ER and/or PR were positive and HER2 negative, HER2+ when HER2 was positive, and TN when the three receptors were negative.

We also included NGS and aCGH data of metastatic samples from 468 non‐IBC patients pooled from our PERMED‐01 prospective clinical trial (NCT02342158) (*N* = 174) and from two public sets: Lefebvre *et al. *(2016) (*N* = 216) and the Metastatic Breast Cancer Project (2018) (*N* = 78). Moreover, we used the gene expression data from the International IBC Consortium (137 IBC and 252 non‐IBC samples) (Van Laere *et al.*, 2013) to apply gene expression signatures of NOTCH (Villanueva *et al.*, 2012) and E2F4 (Guerrero‐Zotano *et al.*, 2018) activation.

### 2.2 DNA copy number profiling

In three series (IPC, TCGA, Metabric), the DNA copy number profiles were established by using whole‐genome aCGH: high‐resolution 4 × 180K CGH microarrays (SurePrint G3‐Human CGH‐Microarray; Agilent Technologies, Massy, France) for IPC (Bertucci *et al.*, 2016), and Affymetrix single nucleotide polymorphisms (SNP) 6.0 arrays (Santa Clara, CA, USA) for TCGA and Metabric. All aCGH probes were mapped according to UCSC Build 37 (hg19). In the other series (TCRU, Ross, Hamm), the DNA copy number of tumors was derived from targeted NGS (tNGS) data generated by Foundation Medicine. The CNA results of those public sets were collected as processed data from the GDC Data Portal for the TCGA series, cBioPortal for Metabric, and the journal websites for Ross and Hamm series. Across all series, we used one threshold value (log_2_ ratio > |1|) to define amplifications and deletions. The homologous recombination deficiency score (HRD) (Marquard *et al.*, 2015) was defined on segmented data processed with circular binary segmentation and considered positive above 10 (Olshen *et al.*, 2004). We searched for chromothripsis in IBC by applying the CTLPScanner (Yang *et al.*, 2016).

### 2.3 Mutational profiling

All series were sequenced using Illumina platforms. Except the TCGA series, which used WES, the other ones used tNGS. IPC samples were sequenced with a home‐made panel of 493 ‘cancer‐associated’ genes (CCP‐V8 panel, Table S2). The DNA libraries of all coding exons and intron–exon boundaries of all genes were prepared using the HaloPlex Target‐Enrichment‐System (Agilent, Santa Clara, CA, USA) as described (Bertucci *et al.*, 2016), and sequencing was done using the 2 × 150‐bp paired‐end technology on the NextSeq500 Illumina platform (Illumina, San Diego, CA, USA). All sequence data were aligned to UCSC hg19 and analyzed as described (Bertucci *et al.*, 2016). Pathogenicity scores for the single nucleotide variant (SNVs) were obtained with Annovar. Mutations were classified as ‘neutral’ or ‘damaging’ using the majority rule of predictor softwares (provided by dbnsfp: Sift, Polyphen2, LRT, MutationTaster, MutationAssesor, FATHMM, RadialSVM, LR). The TCRU, Ross's and Hamm's series were sequenced by Foundation Medicine (Cambridge, MA, USA) for, respectively, 324, 195/255, and 225 genes. The Metabric series (Pereira *et al.*, 2016) was analyzed on a 173‐gene panel. Sequencing data of the public sets and TCRU were collected and processed as indicated above. The TMB was defined as the number of nonsilent mutations per megabase (Mb) of genome sequenced (Bertucci *et al.*, 2016).

### 2.4 Definition of actionable gene alterations

We defined the AGAs by using the Perera‐Bel's algorithm (Perera‐Bel *et al.*, 2018), which matches patient‐specific genomic alterations to treatment options. This model is based upon public knowledge of somatic variants with predictive evidence on drug response. It is based upon several public data including Gene‐Drug‐Knowledge‐database (GDKD), Clinical‐Interpretation‐of‐Variants‐in‐Cancer (CIViC), and Tumor‐Alterations‐Relevant‐for‐Genomics‐driven‐Therapy (TARGET). The molecular alterations of 312 actionable genes are classified into a six‐level system to rank the associations according to their evidence. The system uses two axes representing the cancer‐type (axis A/B) and the strength of clinical evidence (axis 1/2/3). Levels A and B mean evidence in the same cancer‐type (here breast cancer) and in any other cancer‐type, respectively. Level‐1 means supported by drug approval organizations/clinical guidelines, level‐2 contains clinical evidence, in which late clinical trials are ranked higher followed by early clinical trials and case reports, and level‐3 consists of preclinical evidence. The highest level is A1, followed by B1, then A2, B2, A3, and B3. Our analysis was limited to alterations noted as associated with ‘sensitivity’ to drugs or ‘response’.

### 2.5 Statistical analysis

Correlations between tumor classes and clinicopathological and molecular variables were analyzed using Student's *t*‐test or Fisher's exact test when appropriate. Uni‐ and multivariate analyses comparisons of the frequency of molecular alterations between the tumor groups adjusted for the molecular subtypes and the AJCC stage were done using logit link function. Genes with *p*‐value inferior to 0.05 and *q*‐value inferior to 0.2 in uni‐ and multivariate analyses were considered as significant. Ontology analysis (DAVID database: https://david.ncifcrf.gov/) of the gene list was limited to the Reactome pathways. Hypergeometric test assessed the significance of enrichment of genes common to the different gene lists. The significance of the *P*‐values threshold was set at 5% and analyzes used the R‐software (version 2.15.2: http://www.cran.r-project.org/).

## 3 Results

### 3.1 Population and genes analyzed

We analyzed 101 IBCs and 2351 non‐IBCs (Table 1). As expected, IBCs were associated with more unfavorable prognostic features than non‐IBCs: younger age, prevalent ductal type, higher AJCC stage (including stage 4), higher pathological grade, and more frequent HER2+ and TN subtypes. Forty percent of samples were TN, and 60% were non‐TN in IBC, *vs* 17% and 83%, respectively, in non‐IBC. By definition, all IBC were stage 3 or 4, but the precise stage (3 or 4) was available for 59/101 cases, including 33 stage 3 (59%) and 23 stage 4 (41%). Across all six data sets included, there were five different targeted gene panels and one whole‐exome. The CCP‐V8 panel gene list was compared with the four other lists retrieved from the Foundation Medicine website for TCRU, Ross and Hamm series, and the journal website for Metabric. Because there were only 41 genes common to all panels, we focused our analysis on 756 different genes defined as being present in at least one targeted panel (Table S2).

**Table 1:** Clinicopathological characteristics of patients and samples.

Characteristics	*N*	All cases	Type		*P*‐value
			non‐IBC	IBC	
Age	2397	59.75 (24–96.29)	60 (26–96.29)	49.5 (24–80)	2.43E‐07
Pathological type					
Ductal	1808	1808 (76%)	1763 (75%)	45 (98%)	2.61E‐03
Lobular	277	277 (12%)	276 (12%)	1 (2%)	
Other	309	309 (13%)	309 (13%)	0 (0%)	
Pathological grade					
1	146	146 (11%)	146 (11%)	0 (0%)	1.02E‐03
2	509	509 (38%)	498 (39%)	11 (25%)	
3	682	682 (51%)	649 (50%)	33 (75%)	
AJCC stage					
1–2	1615	1615 (82%)	1615 (87%)	0 (0%)	< 1.00E‐06
3–4	349	349 (18%)	248 (3%)	101 (100%)	
ER status					
Negative	587	587 (25%)	540 (23%)	47 (64%)	3.44E‐13
Positive	1791	1791 (75%)	1765 (77%)	26 (36%)	
PR status					
Negative	1069	1069 (45%)	1017 (44%)	52 (72%)	2.94E‐06
Positive	1305	1305 (55%)	1285 (56%)	20 (28%)	
ERBB2 status					
Negative	1937	1937 (85%)	1891 (85%)	46 (65%)	3.09E‐05
Positive	355	355 (15%)	330 (15%)	25 (35%)	
Molecular subtype					
HR+/HER2−	1520	1520 (66%)	1502 (68%)	18 (25%)	< 1.00E‐06
HER2+	355	355 (16%)	330 (15%)	25 (35%)	
TN	415	415 (18%)	387 (17%)	28 (40%)	

### 3.2 Gene alterations in IBC

**Figure 1:**
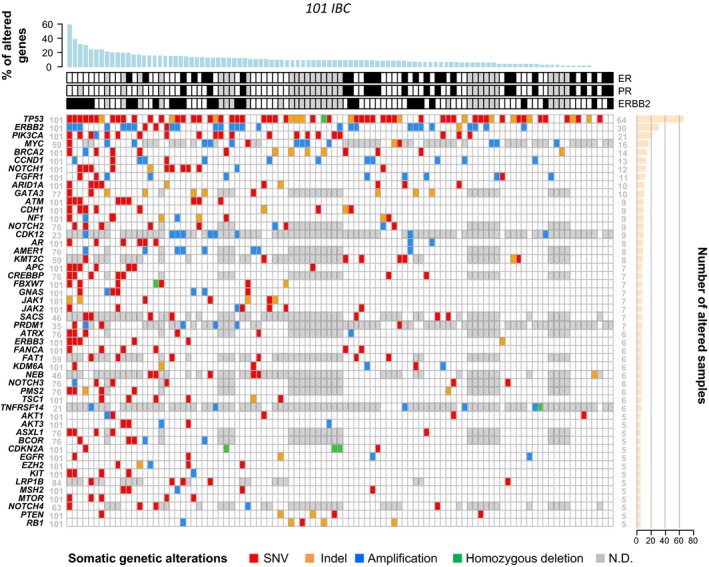
Distribution of alterations of the top 50 genes altered in IBC. Oncoprint of the top 50 genes altered in at least two IBC samples and analyzed in at least 20 samples. Top: immunohistochemical status for ER, PR, and ERBB2 (white: negative; black: positive; gray: unavailable). Bottom: somatic gene alterations (mutations and CNA) color‐coded according to the legend. The genes are ordered from top to bottom by decreasing number of altered tumors (right panel) and the tumors from left to right by decreasing percentage of altered genes (top panel). ND: not defined.

We identified 1101 gene alterations through the 101 IBCs, including 228 amplifications (21% of all alterations), 15 deletions (1%), and 857 mutations (78%), comprising 730 SNVs (nonsynonymous, stop‐gains, splice‐site; 66%), and 127 indels (12%). They corresponded to 1013 different alterations involving 331 different genes (Table S3). The distribution of alterations of the top 50 genes altered in at least two IBCs is shown in Fig. 1. The 10 most frequently altered genes were *TP53* (63%), *HER2* (30%), *MYC* (27%), *PIK3CA* (21%), *BRCA2* (14%), *CCND1* (13%), *GATA3* (13%), *NOTCH1* (12%), *FGFR1* (11%), and *ARID1A* (10%). For *HER2*, there was 93% concordance between the clinical status and the CNA. Ninety‐eight percent of IBC samples (99/101) harbored at least one alteration. The mean number of alterations per sample was 11 (CI95, 9–13). The mean TMB was six mutations per Mb (CI95, 4–8) (Fig. S1). Chromothripsis was present in 20 out of 44 tested IBC (45%). The most affected chromosomes were chromosome 17 (8% of samples), followed by chromosomes 11 (5%) and 8 (3%). The presence of chromothripsis tended to be associated with the molecular subtype: 69% of HER2+ samples displayed chromothripsis, *vs* 35% of HR+/HER2− and 30% of TN (*P* = 0.092; Fisher's exact test).

### 3.3 Comparison of gene alterations between IBC and non‐IBC

Similar analysis was done in the 2351 non‐IBCs. We identified 22 936 gene alterations, corresponding to 14 448 different alterations (Table S3). The distribution of the types of alterations was different from that of IBC (*P* = 1.24E‐17, Fisher's exact test) with a lesser percent of mutations (70% *vs* 78%, corresponding to 62% *vs* 66% for SNVs, and 8% *vs* 12% for indels). The gene alterations identified in non‐IBC confirmed the literature data (Banerji *et al.*, 2012; Ellis *et al.*, 2012; Ferrari *et al.*, 2016; Nik‐Zainal *et al.*, 2012a; Nik‐Zainal *et al.*, 2016; Nik‐Zainal *et al.*, 2012b; Shah *et al.*, 2012; Stephens *et al.*, 2012; Cancer Genome Atlas, 2012), that is, the most frequently altered genes including *PIK3CA* (39%), *TP53* (34%), *HER2* (13%), *GATA3* (13%), *KMT2C* (11%), *CDH1* (10%), and *MAP3K1* (10%). The mean TMB for all variants was higher in IBC (six mutations/Mb; CI95, 4–8) than in non‐IBC (2; CI95, 2–2; Student's *t*‐test, *P* = 6.29E‐05; Fig. S1). Sixteen percent of IBC samples presented a TMB > 10 *vs* only 1% of non‐IBC samples (*P* = 3.36E‐12, Fisher's exact test). The same difference was observed when SNVs and indels were analyzed separately (Fig. S1), and all those differences persisted in multivariate analysis (MV) adjusted for the molecular subtypes, the type of NGS (targeted *vs* WES), and the AJCC stage.

We then applied similarly adjusted supervised analysis to search for genes with differential frequency of alterations between IBC and non‐IBC. Of note, when a sample was not informative for the gene tested, it was excluded from analysis. We identified 96 genes differentially altered (*p* < 0.05 and *q* < 0.20 in both univariate and multivariate analyses), including 95 more frequently altered in IBC and only one (*PIK3CA*) more frequently altered in non‐IBC (Table S4).

**Figure 2:**
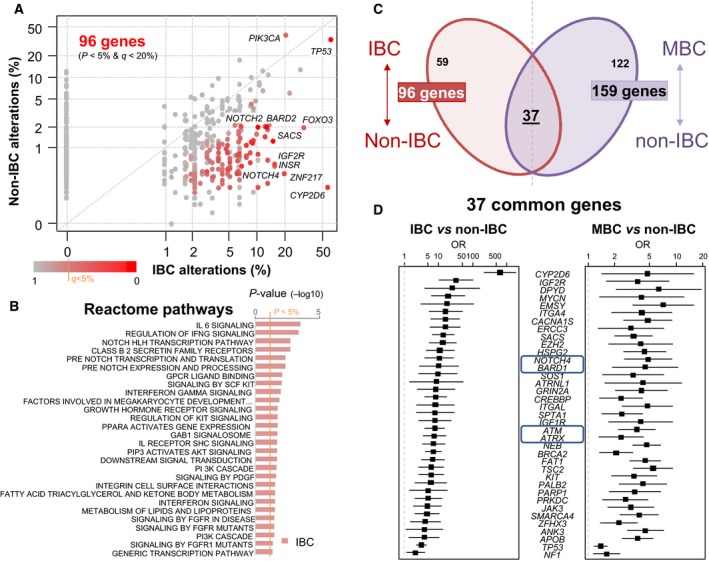
Identification of genes with differential frequency of alterations between samples. (A) Scatter plot depicting the alteration frequency (% of patients) between IBC and non‐IBC. Each dot represents one gene, and dots are color‐coded according to the *P*‐values (−log_10_ *P*‐values) according to the legend below. Significantly mutated genes in either IBC or non‐IBC are included. A few genes differentially mutated are labeled. (B) Ontology analysis revealed several Reactome pathways significantly associated with the 95 IBC genes. (C) Crossings of the lists of genes differentially altered in IBC *vs* non‐IBC (96 genes) and of genes differentially altered in metastatic (MBC) *vs* primary non‐IBC (159 genes). (D) List of 37 genes common to the two gene lists. OR: odds ratio of frequencies of alterations in the tumor subgroups.

The most differentially altered gene was *CYP2D6.* Four genes (*CYP2D6*, *FOXO3*, *TP53*, and *ZNF217)* were altered in > 20% of IBCs and 57 genes such as *BRCA2*, *ATM*, *ATRX*, *EMSY*, *NOTCH2*, and *NOTCH4* were altered in 5–20% of cases. Ontology analysis of the 96 differential genes revealed several pathways associated with IBC genes, such as NOTCH‐related pathways, interleukins and interferon signal, and KIT signaling (Fig. 2B). Genes involved in chromatin remodeling were also more frequently altered in IBC, such as *EZH2* and *SMARCA4*, altered in 5% of IBC, providing a rationale for the evaluation of epigenetic modifiers for the treatment of IBC. Of note, the use of the PAM50‐based genomic definition of molecular subtypes and the use of the IHC definition applied to the 1773 samples (41 IBC and 1732 non‐IBC) informative for both definitions and for the MV showed similar results with the two definitions: 54 and 51 genes were identified as differential with the IHC definition and the PAM50 definition, respectively, with 49 (91% and 96%, respectively) common genes (Fig. S2).

Supposing that these 96 differentially altered genes might be related to IBC aggressiveness, we tested whether they were also differentially altered in metastatic *vs* primary non‐IBC. We compared the frequency of alterations between 468 metastatic samples of non‐IBC patients and the 2351 non‐IBC primary samples. By using the same significance threshold as above, we found 159 differentially altered genes, most of them being more frequently altered in metastatic samples (Table S5). The comparison with the above‐quoted 96‐gene list identified 37 genes more frequently altered in both IBC *vs* non‐IBC samples and in metastatic *vs* primary non‐IBC samples (Fig. 2C,D). Such overrepresentation was significant (*P* = 5.58E‐06, hypergeometric test) and indirectly validated the association of our 96‐gene list with IBC, known for its stronger metastatic potential than non‐IBC. These 37 common genes included genes involved in DNA repair (*ATM*, *ATRX*, *BARD1, BRCA2, EMSY, PALB2*) and in NOTCH pathway (*NOTCH4*). By contrast, the same analysis between the 468 metastatic samples of non‐IBC patients and the 101 IBC samples identified only one gene differentially altered (*HER2*), indicating that IBC and metastatic non‐IBC samples are not so different at a genomic level when compared head‐on.

### 3.4 Actionable genetic alterations in IBC *versus* non‐IBC

**Figure 3:**
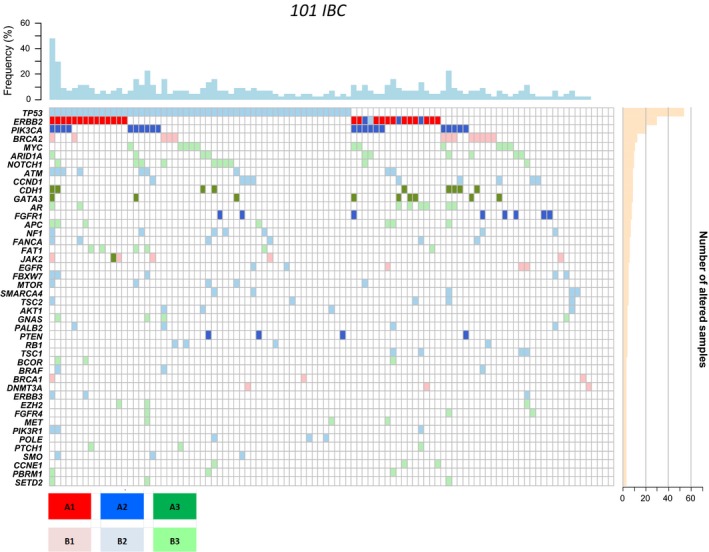
Distribution of genes with actionable alterations in IBC. The 44 genes with actionable alterations in at least four IBC are shown. The genes are ordered from top to bottom by decreasing frequency of mutations. The degree of evidence of actionable alterations according to the Perera‐Bel's algorithm (2018) is color‐coded as indicated in the color scale.

We assessed the distribution of AGAs in IBC, comparatively to non‐IBC, using the Perera‐Bel's algorithm (Perera‐Bel *et al.*, 2018). The percentage of IBC patients with AGAs was high (97%) with 26% of A1 alterations, which corresponded to *HER2* amplification, 24% of B1, 18% of A2, and 29% of B2 (Table S3). Examples of B1 alterations included *BRCA2*, *JAK2,* and *EGFR* alterations observed in 13 (13%), five (5%), and three (3%) patients, respectively. Examples of A2 alterations included *PIK3CA, FGFR1,* and *PTEN* alterations observed in 21 (21%), eight (8%), and four (4%) patients, respectively. Examples of B2 alterations included *CCND1* and *ATM* (nine cases each: 9%), *NF1* (seven cases: 7%), *MTOR* and *TSC2* (five cases each: 5%)*, AKT1*, *RB1*, and *TSC1* (four cases each: 4%), and *ERBB3* (three cases: 3%). Figure 3 shows the distribution of 44 genes with AGA in at least four IBC samples. The most frequent actionable targets with evidence‐level between A1 and B2 were *TP53*, *HER2*, *PIK3CA*, *BRCA2*, *CCND1*, *FGFR1*, *ATM*, and *NF1*. Many samples had several AGAs simultaneously. This percentage of patients with AGAs was higher than the one observed in non‐IBC (87%; *P* = 4.65E‐20, logit link; Fig. S3A), and the difference remained significant in MV (*P* = 5.65E‐14, logit link). There were significantly more A1 and B1 alterations in IBC and more A2 and A3 alterations in non‐IBC (Fig. S3B).

### 3.5 Enrichment of actionable genetic alterations for different therapeutic classes

We analyzed whether there was enrichment in patients with AGAs in IBC *vs* non‐IBC in specific drug classes and functional pathways (Fig. S4). Regarding the class of PI3K/AKT/mTOR inhibitors, the percentage of patients with AGAs was higher in non‐IBC patients (52% *vs* 40%; *P* = 1.97E‐02), but this difference disappeared in MV (*P* = 0.185). The percentage of ‘actionable patients’ in the class of HER/EGFR inhibitors was higher in IBC (36% *vs* 23% in non‐IBC, *P* = 2.68E‐03) and tended to be significant in MV (*P* = 0.091). This percentage regarding the class of other tyrosine kinase receptors inhibitors, higher in IBC (27% *vs* 18% in non‐IBC) in univariate analysis (*P* = 2.01E‐02), but did not remain significant in MV (*P* = 0.565). The same was observed regarding the class of CDK inhibitors with higher percentage of patients with AGAs in IBC (29% *vs* 15%, *P* = 4.48E‐04), not significant in MV (*P* = 0.180). We applied an E2F4 activation 24‐gene signature associated with sensitivity to the palbociclib CDK4/6 inhibitor and resistance to aromatase inhibitor (Guerrero‐Zotano *et al.*, 2018) to the 389 samples of the International IBC Consortium expression dataset (Fig. S5). The corresponding metagene score was higher in IBC than in non‐IBC samples (*P* = 3.68E‐04, Student's *t*‐test; *P* = 5.93E‐03, Fisher's exact test), and this difference remained independent from the molecular subtypes and the AJCC stage (*P* = 0.055, glm; Fig. S5A). This enrichment concerned the HR+/HER2− subtype, which is currently the subtype candidate for CDK4/6 inhibitors (Fig. S5B).

### 3.6 DNA repair more frequently altered in IBC

**Figure 4:**
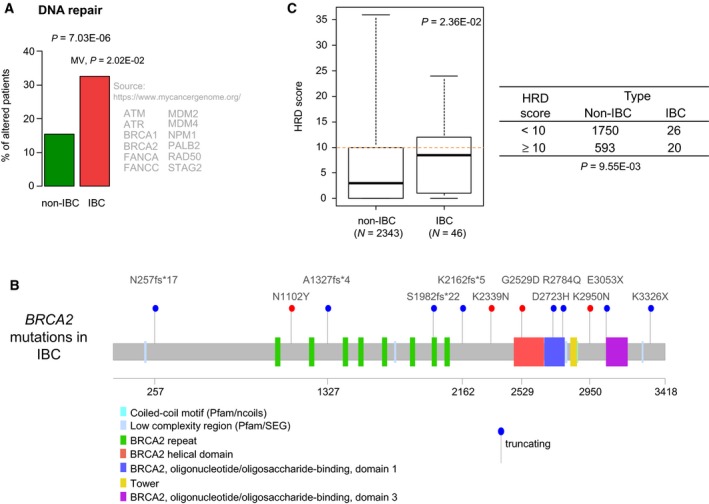
DNA repair genes are more frequently altered in IBC than in non‐IBC. (A) Plot showing the percentage of patients with AGAs in genes involved in DNA repair in IBC *vs* non‐IBC patients. The *P*‐values are for the logit link in univariate analysis and in MV. Beside the plot, are indicated the 12 genes common to the pathway in the indicated bibliographic source and to our list of 756 genes tested. (B) Lolliplot of *BRCA2* gene showing the 12 mutations identified in IBC (blue: truncating mutation; red: nontruncating mutation). (C) Left: Box‐plot of HRD score in non‐IBC and IBC samples. Right: Contingency table between HRD score and IBC/non‐IBC status.

Several genes more frequently altered in IBC such as *ATM*, *ATRX*, *BARD1*, *BRCA2*, and *EMSY* are involved in DNA repair. Pathway analysis confirmed such enrichment: The percentage of patients with alterations of DNA repair genes was 33% in IBC *vs* 17% in non‐IBC (*P* = 7.03E‐06, logit link), even after adjustment in MV (*P* = 2.20E‐02, logit link; Fig. 4A). *BRCA2* was the most frequently altered DNA repair gene in IBC with 13 mutations, including eight truncating mutations (Fig. 4B), suggesting possible enrichment in HRD in IBC. This was confirmed with a higher HRD score in IBCs than in non‐IBCs (*P* = 2.36E‐02, Student's *t*‐test). The OR for high HRD score (≥ 10) was 2.27 (95% CI: 1.19–4.26) in IBC compared with non‐IBC (*P* = 9.45E‐03, Fisher's exact test; Fig. 4C).

### 3.7 NOTCH pathway more frequently altered in IBC

**Figure 5:**
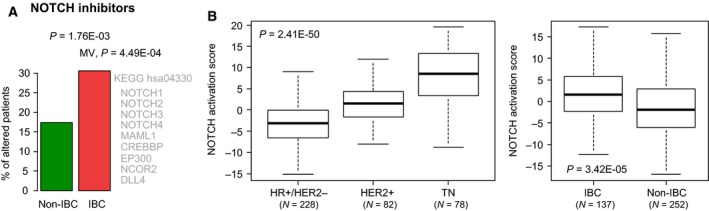
NOTCH pathways genes are more frequently altered in IBC than in non‐IBC. (A) Plot showing the percentage of patients with AGAs involved in the NOTCH pathway in IBC *vs* non‐IBC patients. The *P*‐values are for the logit link in univariate analysis and in MV. Beside the plot, are indicated the nine genes common to the pathway in the indicated bibliographic source and to our list of 756 genes tested. (B) Left: Box‐plot of NOTCH activation score in the breast cancer samples of the International IBC Consortium dataset according to the molecular subtypes. The *P*‐value is for the one‐way ANOVA test. Right: Box‐plot of NOTCH activation score in the breast cancer samples of the International IBC Consortium dataset according to the non‐IBC and IBC statutes. The *P*‐value is for the Student's *t*‐test.

NOTCH pathway alterations were also enriched in patients with IBC (30% *vs* 17% in non‐IBC patients) in univariate (*P* = 1.76E‐03, logit link) and multivariate analyses (*P* = 4.49E‐04, logit link; Fig. 5A). Whereas *NOTCH1* was among the most frequently altered genes in IBC (12%), it was not differentially altered compared with non‐IBC. By contrast, *NOTCH2* and *NOTCH4* were significantly more frequently mutated in IBC with a total of 12 mutations, including nine predicted as damaging *in silico*. There was a mutual exclusivity in IBC samples between alterations in the three genes (*NOTCH2*, *NOTCH4,* and *CREBBP*) found differentially altered in IBC *vs* non‐IBC, present in the KEGG NOTCH pathway, and tested in at least 30% of samples (Fig. S6). Other genes involved in the NOTCH pathway and frequently altered in IBC included *MAML1* (11%), *MED12* (9%), and *FBXW7* (8%).

Such enrichment led to search for signs of NOTCH pathway activation in IBC. We applied a 384‐gene signature of NOTCH activation (Villanueva *et al.*, 2012) to expression data of the International IBC Consortium set. As expected (Shen *et al.*, 2017), the activation score was higher in the TN samples than in the HR+/HER2− samples (*P* = 2.41E‐50, one‐way ANOVA test Fig. 5B), validating its robustness. Interestingly, this score was higher in IBCs than in non‐IBCs (*P* = 3.42E‐05, Student's *t*‐test; Fig. 5B), and this difference remained significant in MV (Table 2).

**Table 2:** Uni‐ and multivariate analyses for IBC *vs* non‐IBC. OR, odds ratio.

	Univariate			Multivariate		
	*N*	OR (95% CI)	*P*‐value	*N*	OR (95% CI)	*P*‐value
Villanueva's NOTCH activation score	389	1.06 (1.04–1.09)	9.27E‐05	384	1.07 (1.03–1.12)	5.82E‐03
Molecular subtype, HER2+ *vs* HR+/HER2−	388	2.81 (1.81–4.36)	1.04E‐04	384	1.40 (0.79–2.48)	0.336
TN vs HR+/HER2‐TN	388	1.96 (1.25–3.08)	1.36E‐02	384	0.77 (0.39–1.53)	0.532
AJCC stage, 3–4 *vs* 1–2	389	1.06 (1.04–1.09)	9.27E‐05	384	4.8E8 (0.00–Inf)	0.981

### 3.8 Comparison with literature

Finally, we compared our results to those of two tNGS studies for which data, publicly unavailable, were not included in our analysis. The Liang *et al.* (2018) study analyzed 91 genes in a series of non‐pretreated primary tumors including 156 IBC and 197 stage 3–4 TCGA non‐IBC: Seventeen genes were more frequently mutated in IBC than in non‐IBC, including *TP53*, *NOTCH2*, *MYH9*, *BRCA2*, *ERBB4*, *POLE*, *FGFR3*, *ROS1*, *NOTCH4*, *LAMA2*, *EGFR*, *BRCA1*, *TP53BP1*, *ESR1*, *THBS1*, *CASP8,* and *NOTCH1*, and one gene, *CDH1*, more frequently mutated in non‐IBC. The Matsuda *et al.* series analyzed 50 genes in non‐pretreated primaries and pretreated relapses of 24 IBC and 376 non‐IBC (Matsuda *et al.*, 2017): Two genes (*TP53*, *HER2*) were more frequently mutated in IBC. In both studies, the comparison was not stratified upon the molecular subtypes.

We compared these lists of differential genes with ours (Fig. S7). Between the Liang *et al.* list and ours, 84 genes were common to both panels tested, with only five differential genes in common: *BRCA2*, *NOTCH2, NOTCH4, POLE,* and *TP53*. Between the Matsuda *et al.* list and ours, 50 genes were common to both panels tested, with only one differential gene (*TP53*) in common. Thirty genes were common to the Matsuda *et al.* and Liang *et al.* panels, with only one differential gene in common: *TP53*. These results revealed low concordance between all three gene lists.

## 4 Discussion

We compared the DNA copy number and mutational profiles of untreated primary tumors of 101 IBCs and 2351 non‐IBCs. Ninety‐seven percent of IBCs displayed at least one AGA. This percentage, higher than in non‐IBC, suggests that personalized therapy is a relevant approach for this aggressive disease, in particular with drugs targeting the DNA repair and NOTCH pathways.

We focused our study on untreated primary tumors to avoid biases induced by previous systemic treatments that induce changes in mutations and subclonal structure between primary tumor and relapses (Bertucci *et al.*, 2019; McGranahan *et al.*, 2015). The scarcity of IBC and diagnostic samples, and the need to adjust analyses upon the molecular subtypes and AJCC stage because of the unbalance between IBC and non‐IBC led us to pool our own bicentric data with available public data. As expected for breast cancers, the genomic profiles were heterogeneous in IBC. We found higher TMB in IBC compared to non‐IBC, possibly related to the higher genomic instability and complexity of the disease (Bekhouche *et al.*, 2011), suggesting that immune checkpoint inhibitors warrant further investigation in IBC (Bertucci *et al.*, 2015; Bertucci and Goncalves, 2017; Van Berckelaer *et al.*, 2019; Van Laere *et al.*, 2013). Such difference was independent from the molecular subtypes and the disease stage. Chromothripsis was identified in 45% of 44 tested IBC, a percentage close to that previously reported in a series of 28 non‐IBC (Przybytkowski *et al.*, 2014).

The 10 most frequently altered genes in IBC are *TP53 *(*63*%)*, HER2 *(*30*%)*, MYC *(*27*%)*, PIK3CA *(*21*%)*, BRCA2 *(*14*%)*, CCND1 *(*13*%)*, GATA3 *(*13*%)*, NOTCH1 *(*12*%)*, FGFR1 *(*11*%)*, and ARID1A *(*10*%), which are also altered in non‐IBC. But the comparison with non‐IBC identified 95 genes more frequently altered in IBC in a molecular subtype and stage‐independent way, including 37 that were also more frequently altered in metastases *vs* primary tumors of non‐IBC patients. This suggests a possible link of these genes with disease aggressiveness and proclivity to metastasize, although functional studies are warranted. Interestingly, the pairwise comparisons of IBC, non‐IBC primaries, and non‐IBC metastatic samples showed that IBC and metastatic non‐IBC are much less different at a genomic level when compared head‐on than are IBC *vs* non‐IBC primaries and primary *vs* metastatic non‐IBC. *CYP2D6* was the most differentially altered gene (58% of IBC *vs* 0.2% in non‐IBC). This gene codes for the cytochrome P‐450 2D6, which oxidizes tamoxifen to its most active metabolite. Many *CYP2D6* polymorphisms, such as the one found in our series (435T>S), have been identified, leading to the decrease of CYP2D6 enzymatic activity. Several data suggest that poor metabolizers of CYP2D6 do not benefit as much from tamoxifen therapy as other patients do; however, conflicting results were published (Hoskins *et al.*, 2009). Absence of analysis of constitutional DNA impedes us to conclude on the SNP nature of our variant. However, the large difference in frequency with non‐IBC potentially reveals an important role for this variant in the predisposition to IBC and/or the known resistance of the disease to standard hormone therapy. Analysis of a larger series is needed, including both tumor and matched normal DNA.

Several therapeutically actionable targets were frequently altered in IBC, including a few ones more frequently than in non‐IBC and independently from the molecular subtypes and AJCC stage. For example, we found frequent alterations in genes involved in DNA repair. *ATM*, *ATRX*, *BARD1*, *BRCA2, ERCC3, MSH2, MSH6, PMS2, and POLE* were more frequently altered in IBC, in which we found also frequent alterations of *TP53*, *FANCA*, and *FANCB*. This observation confirms recent findings (Liang *et al.*, 2018) of frequent alterations in *BRCA1/BRCA2/POLE* genes in IBC. It is likely that deficient DNA repair contributes to disease progression, as well as to the high TMB observed in IBC and indirectly to its peculiar immune microenvironment (Bertucci *et al.*, 2015; Van Berckelaer *et al.*, 2019; Van Laere *et al.*, 2013). In addition, IBC showed more frequently a HRD and alterations in genes involved in mismatch repair, supporting the ongoing development of PARP inhibitors in IBC as radiosensitizers in phase I–II trials with veliparib (Jagsi *et al.*, 2018) and olaparib (NCT03598257) (Michmerhuizen *et al.*, 2019). This observation warrants a deeper NGS study of IBC, using whole‐genome sequencing (WGS) to investigate structural variations. A recent study assessed the prevalence of germline variants in cancer predisposition genes in 368 patients with IBC (Rana *et al.*, 2019). Germline mutations were identified in 53 cases (14.4%). *BRCA1* or *BRCA2* mutations were found in 7.3% of the subjects, 6.3% had a mutation in other breast cancer genes (*PALB2*, *CHEK2*, *ATM*, and *BARD1*), and 1.6% had mutations in genes not associated with breast cancer.

Alterations in the NOTCH pathway were almost twice as enriched in IBC (30% *vs* 17% in non‐IBC), independently from the molecular subtypes and AJCC stage. NOTCH receptors are transmembrane receptors that play an essential role in cell fate decisions, such as proliferation, differentiation, and apoptosis, and in the maintenance of breast cancer stem‐like cells (Mollen *et al.*, 2018). *NOTCH1* was the most frequently altered *NOTCH* gene in IBC (12%), whereas *NOTCH2* and *NOTCH4* were more frequently altered in IBC compared with non‐IBC, with a total of 12 mutations including 9 predicted as damaging. Functional studies measuring NOTCH pathway activation, transformation potential, and sensitivity to pathway inhibition are required to better define the relevance of these alterations. Interestingly, we found a mutual exclusivity in IBCs for *NOTCH2*, *NOTCH4,* and *CREBBP* alterations, present in a total of 19 out of 76 (25%) informative samples. Of note, *NOTCH2* and *NOTCH4* were also reported as more frequently mutated in IBC in the recent Liang's study (Liang *et al.*, 2018). We also found a NOTCH pathway activation score (Villanueva *et al.*, 2012) higher in IBC than in non‐IBC independently from the molecular subtypes and AJCC stage, further supporting a role for the NOTCH pathway in IBC. Several data in literature, based on preclinical models, have also related IBC and NOTCH alterations. In the MARY‐X model, the lymphovascular emboli of IBC exhibit a NOTCH3 addiction (Xiao *et al.*, 2011). The FC‐IBC02 cell line shows *NOTCH3* amplification (Fernandez *et al.*, 2013). Pregnant mice expressing higher levels of an activated intracellular form of NOTCH3 develop luminal mammary tumors resembling IBC that frequently metastasize (Ling *et al.*, 2013). Thus, the NOTCH targeting might be an option for IBC treatment. Accordingly, a preclinical study in IBC showed that a gamma‐secretase inhibitor, RO4929097, was able to block the NOTCH signaling and to attenuate the stem‐like phenotype of IBC cells and regulate the inflammatory environment (Debeb *et al.*, 2012). All this taken together, the NOTCH pathway may constitute the most prominent difference between IBC and non‐IBC.

We also analyzed four other classes of targeted therapies approved or tested in breast cancer. Regarding the PI3K/AKT/mTOR inhibitors, even if *PIK3CA* was the only gene more frequently altered in non‐IBC compared to IBC, its frequency of alteration was relatively high in IBC (21%), with many hotspot mutations. Other actionable genes of the PI3K/AKT/mTOR pathway were frequently altered in IBC with likely loss‐of‐function mutations in *PTEN*, *TSC1*, and *TSC2* and gain‐of‐function mutations in *AKT1*, *AKT3*, *MTOR, RPTOR,* and *RICTOR*. Thus, like non‐IBC patients, IBC patients may benefit from inhibition of the pathway with the PI3K/AKT/mTOR inhibitors approved and in development (Kenna *et al.*, 2018).

Regarding the CDK4/6 inhibitors class, we found twice as higher percentage of patients with AGAs in IBC than in non‐IBC (29% *vs* 15%). The most frequent AGA in this group was *CCND1* amplification (10% of IBCs), followed by *CDKN2A* deletion/mutation (5%). Mutations in the *FAT1* and *RB1* tumor suppressors, potentially associated with resistance to CDK4/6 inhibitors (Li *et al.*, 2018), were also observed in 10% and 4% of IBC, respectively. Interestingly, an E2F4 activation expression signature associated with sensitivity to palbociclib and resistance to aromatase inhibitors (Guerrero‐Zotano *et al.*, 2018) was higher in IBC than in non‐IBC samples, notably in HR+/HER2− patients. Clearly, CDK4/6 inhibitors deserve to be tested in IBC.

The percentage of ‘actionable patients’ in the class of HER/EGFR inhibitors was higher in IBC (36% *vs* 23%), and the difference tended toward significance in MV including the molecular subtypes and AJCC stage. As expected, *ERBB2* amplification was observed in 26% of IBC, and activating *ERBB2* mutations (Petrelli *et al.*, 2017) were much less frequent. Five IBC patients displayed such mutations, including two with (HER2+ patient) and three without (HER2− patient) simultaneous amplification. Mutations were located in the extracellular domain and the kinase domain and have been associated with sensitivity to ERBB2 tyrosine kinase inhibitors such as neratinib (Bose *et al.*, 2013) with which clinical trials are ongoing. Three IBC patients (3%) displayed an *ERBB3* mutation identified as AGA *vs* 42 non‐IBC patients (1.8%). In their small series of cases, Hamm *et al. *(2016) previously reported frequent co‐occurrence of *ERBB3* mutations and *ERBB2* amplification in IBC and suggested possible contribution to resistance to anti‐HER2 therapy. In our larger series, such co‐occurrence was found in 2% of IBC and only 0.2% of non‐IBC, supporting investigation of ERBB3‐inhibitors in combination with ERBB2‐inhibitors in IBC.

## 5 Conclusions

Our study confirms the hypothesis that IBC is distinct from non‐IBC at the genomic level, independently from the molecular subtypes and disease stage. We found higher TMB in IBC than in non‐IBC and 95 genes more frequently altered in IBC in a molecular subtype‐ and stage‐independent way. Ninety‐seven percent of IBC samples displayed at least one AGA. This percentage, higher than in non‐IBC (87%), suggests that precision medicine is a bona fide option in this aggressive disease, notably with drugs targeting DNA repair, NOTCH signaling, and CDK4/6. The strengths of our study are follows: (a) the largest comparison of IBC *vs* non‐IBC samples (total of 2452 samples), and the largest number of genes tested (756 genes) in such comparison, (b) a consensual uniform case definition for IBC, (c) a homogeneous series of non‐pretreated primary tumors, (d) an adjustment upon both the molecular subtypes and AJCC stage, and (e) a statistical correction (FDR with *q*‐values) for multiple tests in the gene‐by‐gene supervised analysis. To our knowledge, these two last points have never been combined in studies comparing the molecular alterations of IBC and non‐IBC. Other strengths include the use of an algorithm to define more objectively the AGAs, the validation of differential activation of potentially targetable pathways (NOTCH, HRD) using transcriptomic and genomic data, and the demonstration that our supervised analysis gave very similar results whatever was the definition of molecular subtypes included in the MV, either IHC‐ or PAM‐based. However, like most of other studies published in the field, it also displays a few limitations: (a) its retrospective nature and associated biases, (b) absence of matched normal DNA sequenced for the tNGS‐based series and absence of information regarding eventual germline mutations and family and personal histories of cancer, (c) presence of missing data for several genes because of the variation in genes targeted across the cohorts examined, leading to a loss of sensitivity regarding identification of genes differentially altered, (d) the use of different sequencing platforms including tNGS and WES, even if that did not impact our comparative analysis as suggested by the MV, and (e) no further analysis of structural variations, driver mutations, intra‐tumor heterogeneity, and mutational signatures. Of course, analysis of a larger and homogeneous series of untreated primary tumors analyzed with WES, WGS, and RNA‐Seq is warranted. Such analysis could also reveal etiology of IBC by identifying DNA sequences not matching to the human genome, such as viral or bacterial infection, as suggested (El‐Shinawi *et al.*, 2016). But yet, our results suggest targeted therapies that have the potential to bring benefit to IBC patients and encourage prospective clinical trials.

## Conflict of interest

The authors declare no conflict of interest.

## Author contributions

Concept, design, and supervision: FB and SVL. Data analysis and interpretation: all authors. Writing and review of manuscript: FB, CR, PF, BD, XW, NU, MCr, DB, SVL. Reading and approval of the final manuscript: all authors.

## Supporting information


**Fig. S1** **.** Tumor mutational burden (TMB) in IBC and non‐IBC.

**Fig. S2** **.** Absence of impact of the definition of molecular subtypes (IHC *vs* PAM50) on the differentially altered character of our 96 genes.

**Fig. S3** **.** Percentage of patients with AGAs along IBC *vs* non‐IBC patients.

**Fig. S4** **.** Percentage of patients with actionable alterations in four specific drug classes.

**Fig. S5** **.** E2F4 activation signature enriched in IBC *vs* non‐IBC.

**Fig. S6** **.** Mutual exclusivity of NOTCH pathway alterations in IBC.

**Fig. S7** **.** Comparison of the lists of genes differentially altered in IBC *vs* non‐IBC across three studies.

**Table S1** **.** IBC and non‐IBC data sets included in the present study.

**Table S2** **.** List of 756 genes analyzed in the present study.

**Table S3** **.** List of gene alterations identified in the 101 IBC and the 2351 non‐IBC.

**Table S4** **.** List of 96 genes differentially altered in IBC *vs* non‐IBC in MV.

**Table S5** **.** List of 159 genes differentially altered in MBC *vs* non‐IBC.

**Table S6** **.** Detailed clinicopathological data and genomic data analyzed in the present study.

## Data Availability

All clinicopathological data and genomic data analyzed in the present study are available in this article in the Table S6.
