# Supplementary material for: NOTCH and DNA repair pathways are more frequently targeted by genomic alterations in inflammatory than in non‐inflammatory breast cancers
Source: Mol Oncol. 2020 Feb 5;14(3):504–19. doi: 10.1002/1878-0261.12621 (PMC7053236; doi:10.1002/1878-0261.12621)
Supplement: Supplementary file 1 — Fig. S1 . Tumor mutational burden (TMB) in IBC and non‐IBC. [file MOL2-14-504-s001.pdf]

Figure S1

A

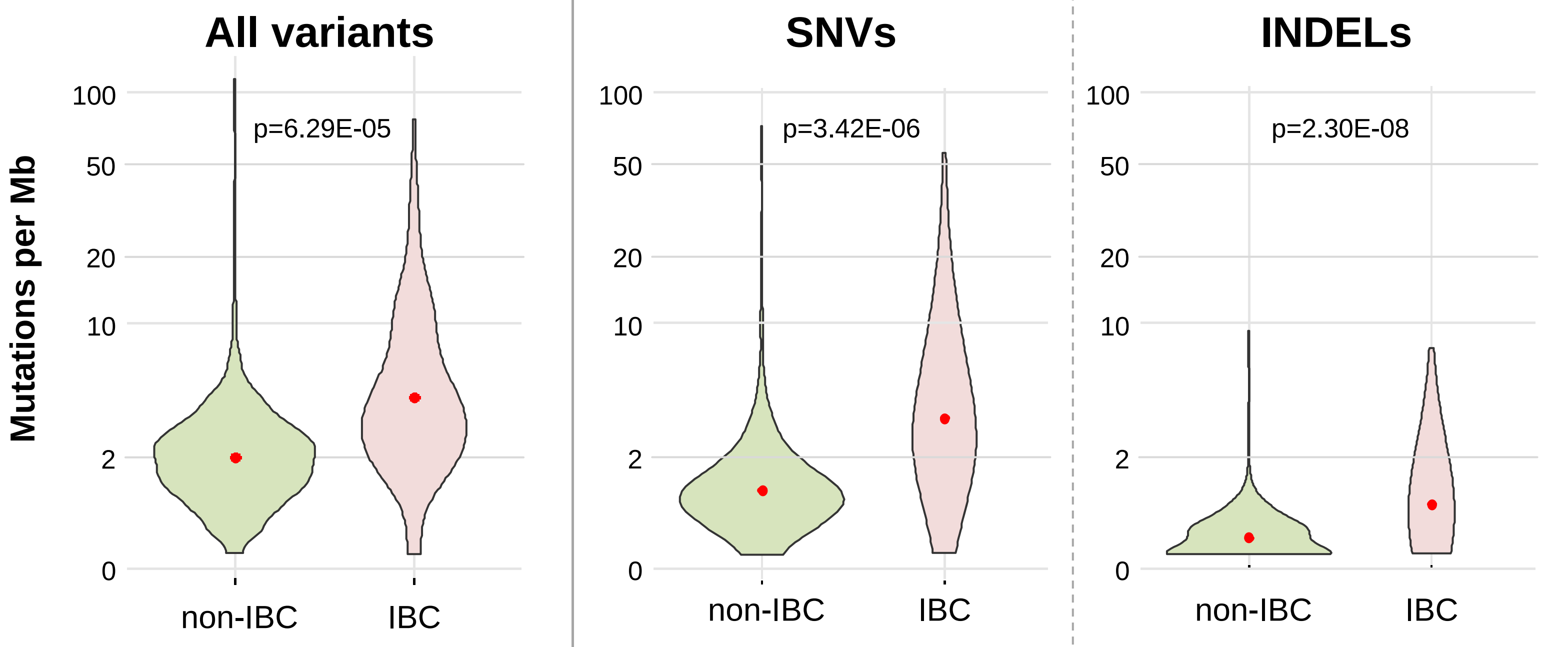

B

TMB	N	Multivariate Odds-ratio [CI95]	p-value	N	Multivariate Odds-ratio [CI95]	p-value	N	Multivariate Odds-ratio [CI95]	p-value
IBC *vs.* nIBC	1797	363.8 [93-1427]	1.82E-12	1817	117.6 [52-267]	2.97E-21	1817	2.03 [1.82-2.26]	2.31E-26
WES *vs.* targeted	1797	1.68 [1.09-2.58]	4.65E-02	1817	2.54 [1.91-3.39]	9.20E-08	1817	1.09 [1.05-1.13]	2.21E-04
HER2+ *vs.* HR+/HER2-	1797	2.15 [1.23-3.76]	2.49E-02	1817	1.89 [1.31-2.75]	4.63E-03	1817	1.02 [0.97-1.07]	5.54E-01
TN *vs.* HR+/HER2-		1.3 [0.76-2.21]	4.16E-01		1.29 [0.91-1.83]	2.37E-01		1.07 [1.02-1.12]	1.44E-02
AJCC stage, III-IV *vs.* I-II	1797	0.76 [0.39-1.45]	4.81E-01	1817	0.83 [0.53-1.28]	4.71E-01	1817	0.96 [0.91-1.02]	2.66E-01
