# Supplementary figures and images for: NOTCH and DNA repair pathways are more frequently targeted by genomic alterations in inflammatory than in non‐inflammatory breast cancers
Source: Mol Oncol. 2020 Feb 5;14(3):504–19. doi: 10.1002/1878-0261.12621 (PMC7053236; doi:10.1002/1878-0261.12621)

Figure S2

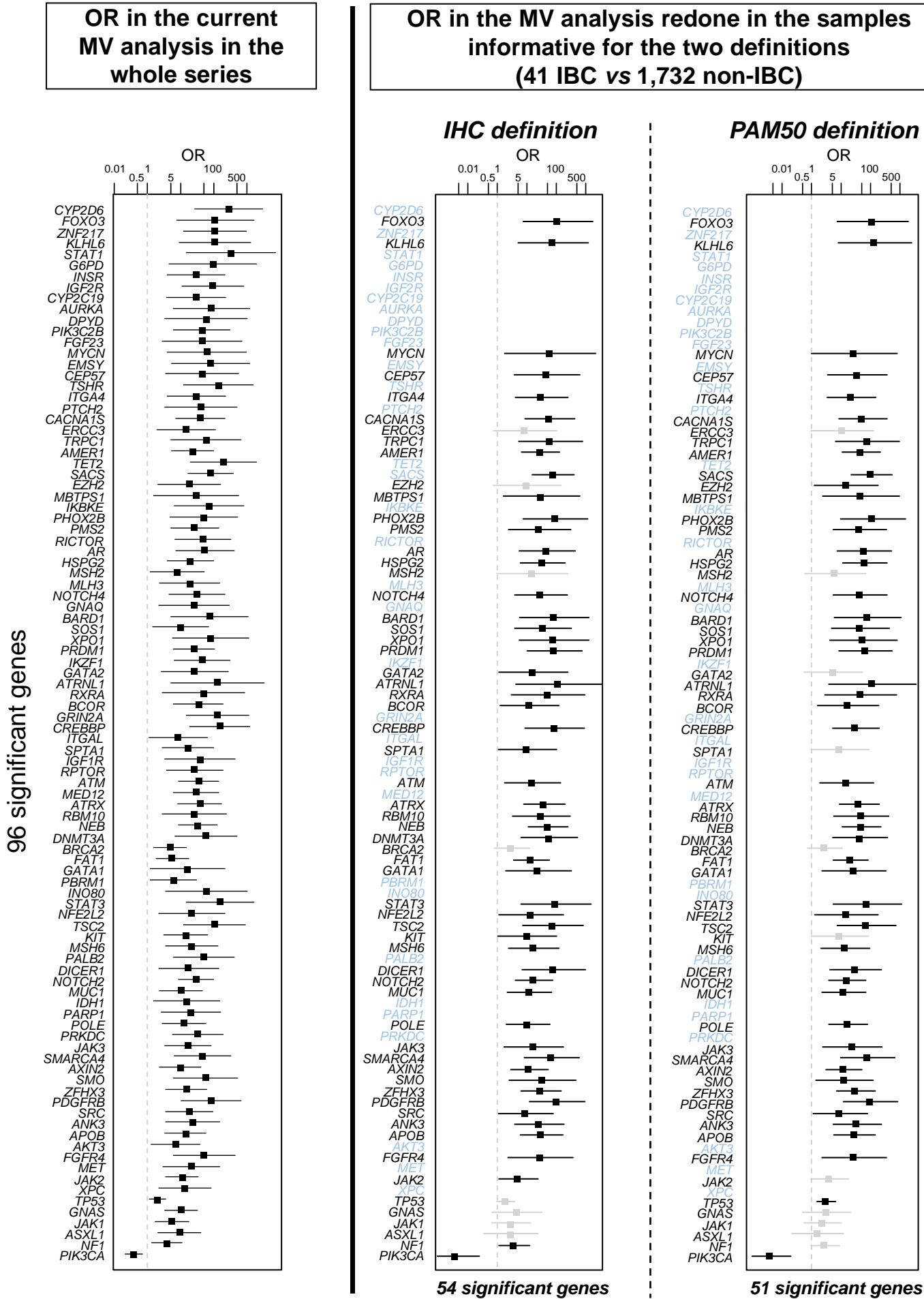

Supplement: Supplementary file 2 — Fig. S2 . Absence of impact of the definition of molecular subtypes (IHC vs PAM50) on the differentially altered character of our 96 genes. [file MOL2-14-504-s002.pdf]

Figure S3

A

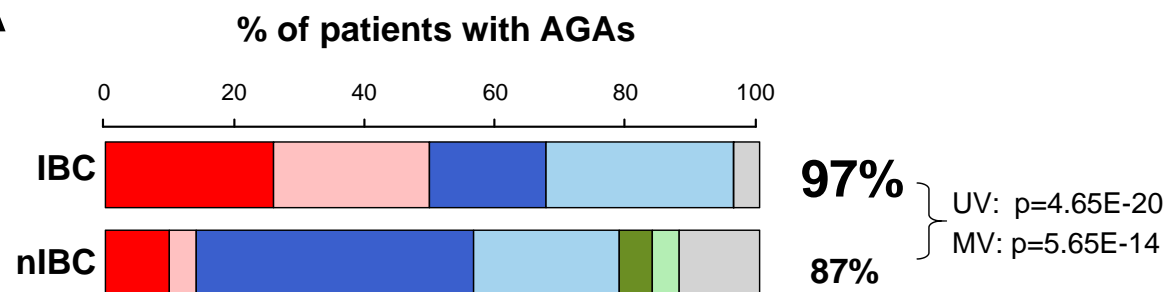

|    |    |    |
|----|----|----|
| A1 | A2 | A3 |
| B1 | B2 | B3 |

B

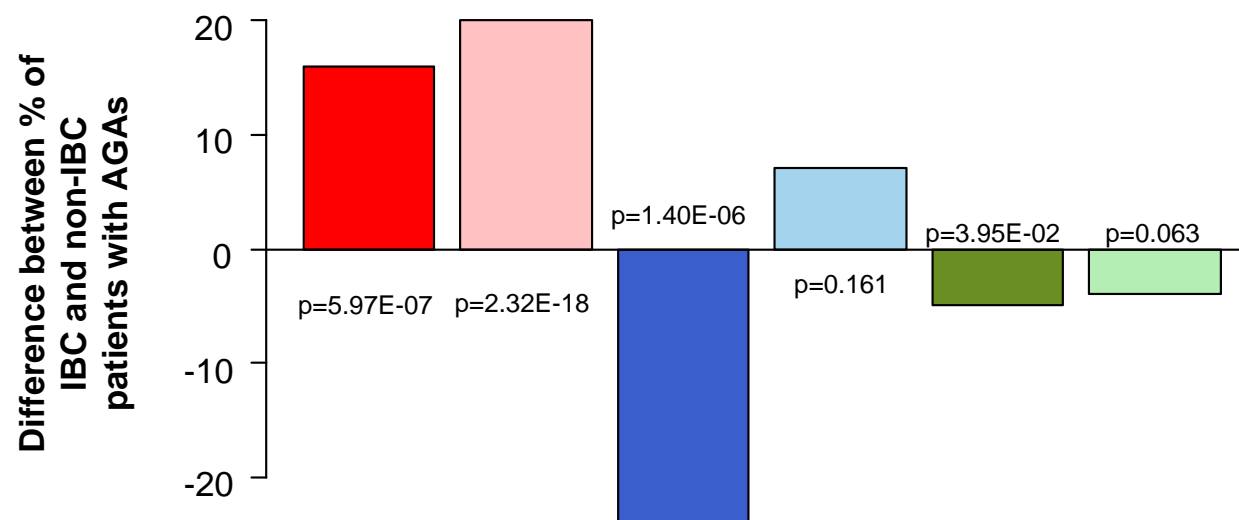

Supplement: Supplementary file 3 — Fig. S3 . Percentage of patients with AGAs along IBC vs non‐IBC patients. [file MOL2-14-504-s003.pdf]

Figure S6

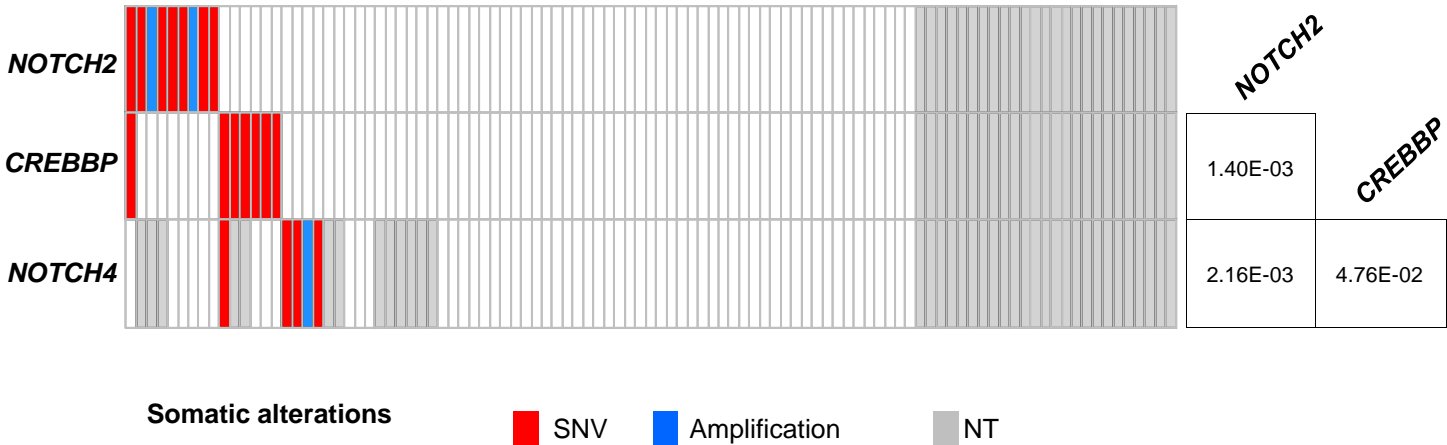

Supplement: Supplementary file 6 — Fig. S6 . Mutual exclusivity of NOTCH pathway alterations in IBC. [file MOL2-14-504-s006.pdf]

Figure S7

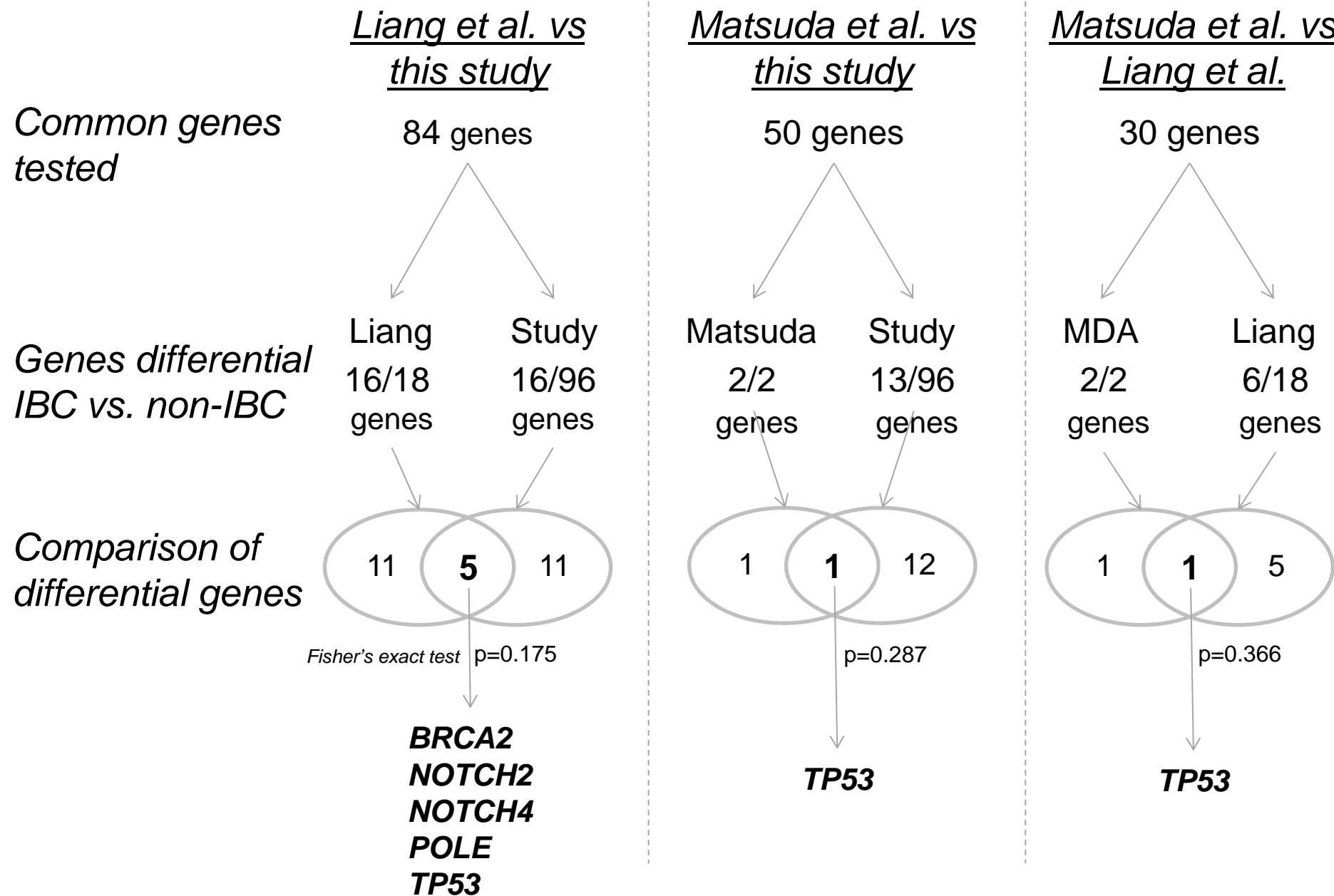

Supplement: Supplementary file 7 — Fig. S7 . Comparison of the lists of genes differentially altered in IBC vs non‐IBC across three studies. [file MOL2-14-504-s007.pdf]
