# Supplementary material for: NOTCH and DNA repair pathways are more frequently targeted by genomic alterations in inflammatory than in non‐inflammatory breast cancers
Source: Mol Oncol. 2020 Feb 5;14(3):504–19. doi: 10.1002/1878-0261.12621 (PMC7053236; doi:10.1002/1878-0261.12621)
Supplement: Supplementary file 4 — Fig. S4 . Percentage of patients with actionable alterations in four specific drug classes. [file MOL2-14-504-s004.pdf]

Figure S4

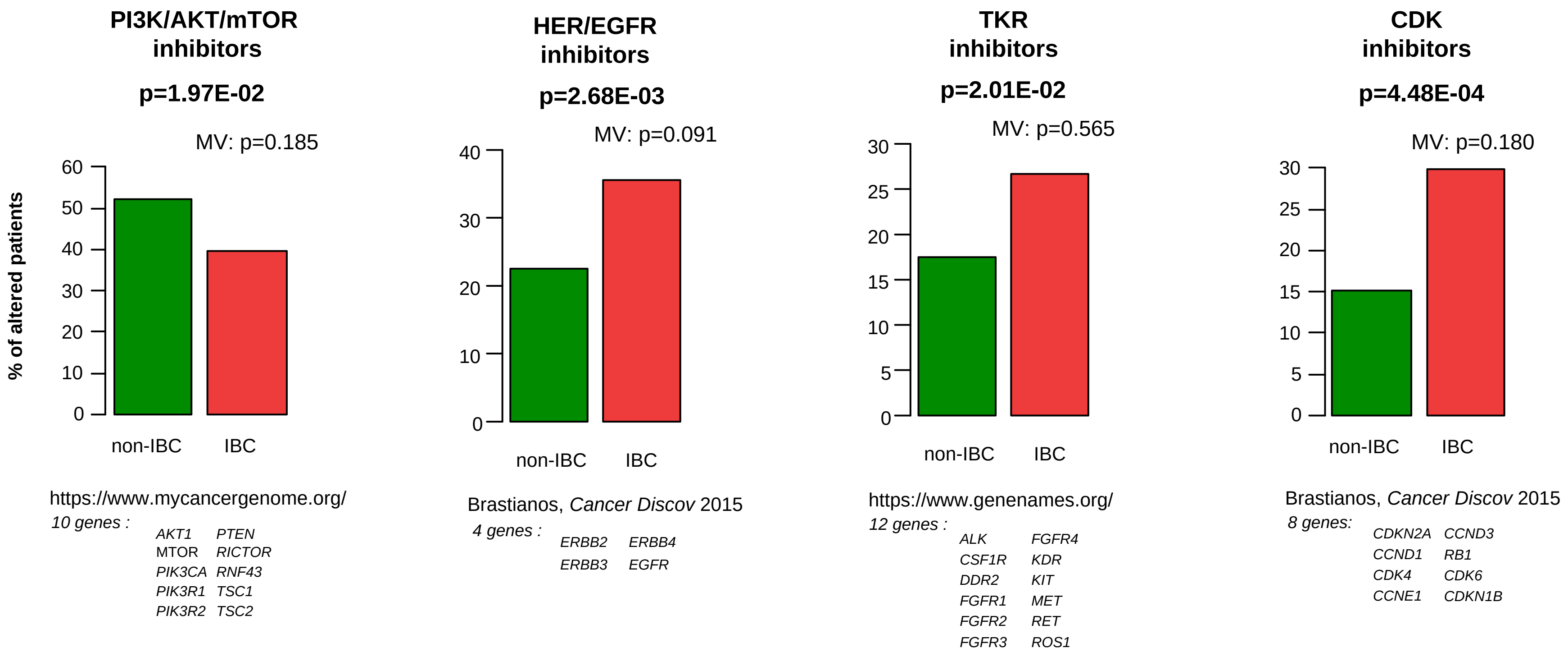
PI3K/AKT/mTOR inhibitors
p=1.97E-02
MV: p=0.185
% of altered patients
0
10
20
30
40
50
60
non-IBC
IBC
https://www.mycancergenome.org/
10 genes :
AKT1 PTEN
MTOR RICTOR
PIK3CA RNF43
PIK3R1 TSC1
PIK3R2 TSC2
HER/EGFR inhibitors
p=2.68E-03
MV: p=0.091
0
10
20
30
40
non-IBC
IBC
Brastianos, Cancer Discov 2015
4 genes :
ERBB2 ERBB4
ERBB3 EGFR
TKR inhibitors
p=2.01E-02
MV: p=0.565
0
5
10
15
20
25
30
non-IBC
IBC
https://www.genenames.org/
12 genes :
ALK FGFR4
CSF1R KDR
DDR2 KIT
FGFR1 MET
FGFR2 RET
FGFR3 ROS1
CDK inhibitors
p=4.48E-04
MV: p=0.180
0
5
10
15
20
25
30
non-IBC
IBC
Brastianos, Cancer Discov 2015
8 genes:
CDKN2A CCND3
CCND1 RB1
CDK4 CDK6
CCNE1 CDKN1B
