# Supplementary material for: NOTCH and DNA repair pathways are more frequently targeted by genomic alterations in inflammatory than in non‐inflammatory breast cancers
Source: Mol Oncol. 2020 Feb 5;14(3):504–19. doi: 10.1002/1878-0261.12621 (PMC7053236; doi:10.1002/1878-0261.12621)
Supplement: Supplementary file 5 — Fig. S5 . E2F4 activation signature enriched in IBC vs non‐IBC. [file MOL2-14-504-s005.pdf]

Figure S5

A

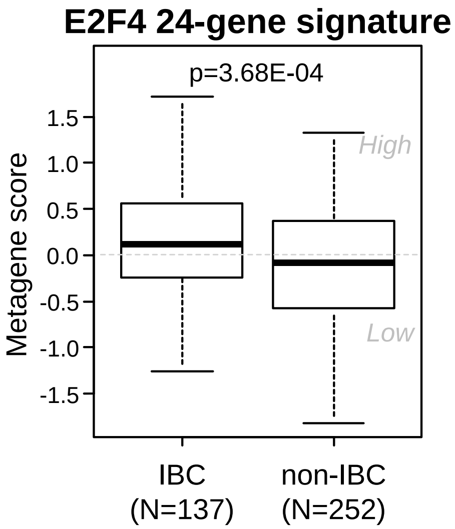

	N	IBC	non-IBC	p-value	Statistic
E2F4 24-g signature				5.93E-03	0.55
low	196	56 (41%)	140 (56%)		[0.35-0.86]
high	193	81 (59%)	112 (44%)		

IBC vs. non-IBC, glm	N	Multivariate OddsRatio [CI95]	pvalue
E2F4 24-g signature	384	0.659 [0.46-0.942]	5.50E-02
Subtype, HER2+ vs. HR+/HER2-	384	0.632 [0.36-1.11]	0.178
TN vs. HR+/HER2-	384	0.868 [0.473-1.59]	0.702
AJCC stage, III-IV vs. I-II	384	2.2E-09 [0e+00-Inf]	0.981

B

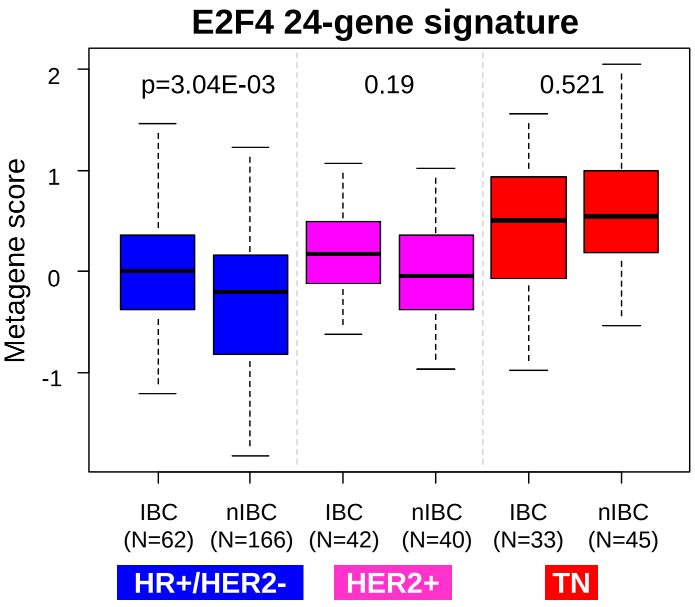

		N	IBC	non-IBC	p-value	Statistic
HR+/HER2-	E2F4 24-g signature				3.35E-02	0.52
	low	140	31 (50%)	109 (66%)		[0.28-0.99]
	high	88	31 (50%)	57 (34%)		
HER2+	E2F4 24-g signature				0.267	0.56
	low	37	16 (38%)	21 (52%)		[0.21-1.46]
	high	45	26 (62%)	19 (48%)		
TN	E2F4 24-g signature				0.588	1.5
	low	18	9 (27%)	9 (20%)		[0.45-4.95]
	high	60	24 (73%)	36 (80%)		
