# Supplementary material for: NOTCH and DNA repair pathways are more frequently targeted by genomic alterations in inflammatory than in non‐inflammatory breast cancers
Source: Mol Oncol. 2020 Feb 5;14(3):504–19. doi: 10.1002/1878-0261.12621 (PMC7053236; doi:10.1002/1878-0261.12621)
Supplement: Supplementary file 14 [file MOL2-14-504-s014.docx]

**Supporting Information**

**Supplementary Figure 1:** **Tumor mutational burden (TMB) in IBC and non-IBC.**

**A/** Violin plots showing the distribution of the TMB in IBC and non-IBC. The left plots correspond to all variants, the middle plots to SNVs only, and the right plots to indels only. The p-values are for the Student t-test. **B/** Multivariate analyses for TMB including the IBC/non-IBC phenotype, the sequencing platform (WES/targeted NGS), the molecular subtypes, and the AJCC stage. The left analysis correspond to all variants, the middle analysis to SNVs only, and the right analysis to indels only.

**Supplementary Figure 2: Absence of impact of the definition of molecular subtypes (IHC *versus* PAM50) on the differentially altered character of our 96 genes.**

Plots showing the Odds Ratios (OR) of each of the 96 genes (identified as differentially altered between IBC and non-IBC) observed in the multivariate analysis. The genes are ordered from top to bottom according to their decreasing OR observed in the univariate analysis done in the whole series. The *left* panel is for the whole series of samples; the *middle* and *right* panels are for the 1,773 samples (41 IBC *vs* 1,732 non-IBC) informative for both the IHC definition and the PAM50 definition of molecular subtypes and for the variables included in the multivariate analysis: the middle panel shows the results using the IHC definition and the right panel shows the results using the PAM50 definition. The genes written in blue correspond to the 34 genes that became statistically not assessable because of the low incidence of gene alterations, leaving 62 genes (written in black) statistically assessable. All these 62 genes displayed an OR in the same direction (positive or negative) as in the whole series of samples, and 54 (87%) remained significant with the “clinical” IHC definition and 51 (82%) remained significant with the “molecular” PAM50 definition. And, 49 genes were common to the 54 (91%) and 51 (96%) genes, suggesting that the definition of subtype did not modify the results of our IBC *versus* non-IBC analysis.

**Supplementary Figure 3:** **Percentage of patients with actionable genetic alterations along IBC *versus* non-IBC patients.**

**A/** Percentage of patients with AGAs as defined according to the Perera-Bel’s algorithm in IBC and non-IBC. UV: univariate logistic regression analysis. MV: multivariate analysis. **B/** Difference of percentage of patients with AGAs between IBC and non-IBC.

**Supplementary Figure 4:** **Percentage of patients with actionable alterations in four specific drug classes.**

Plots showing the percentage of patients with AGAs along IBC *versus* non-IBC patients in four specific drug classes: PI3K/AKT/mTOR inhibitors, HER/EGFR inhibitors, TKR inhibitors, and CDK inhibitors. The p-values are for the Logit link in univariate analysis and in multivariate analysis (MV). Below each drug class, are indicated the genes common to the pathway in the indicated bibliographic source and to our list of 756 genes tested.

**Supplementary Figure 5:** **E2F4 activation signature enriched in IBC *versus* non-IBC.**

**A/** *Left*: box-plot of E2F4 activation 24-gene metagene n the 389 breast cancer samples of the International IBC Consortium data set; the p-value is for the Student t-test. *Right and top*: cross-table between the IBC/non-IBC phenotype and the activation status (low/high) according to the E2F4 signature; the p-value is for the Fisher’s exact test. *Right and bottom*: multivariate analysis for IBC versus non-IBC phenotype integrating the E2F4 activation signature, the molecular subtypes and AJCC stage. **B/** Similar to A, but in each molecular subtype separately.

**Supplementary Figure 6: Mutual exclusivity of NOTCH pathway alterations in IBC.**

Pattern of molecular alterations of NOTCH pathway genes in IBC (mutual exclusivity p-value < 0.05, Fisher’s exact test); NT, not tested.

**Supplementary Figure 7:** **Comparison of the lists of genes differentially altered in IBC *versus* non-IBC across three studies.**

Comparison of the lists of genes differentially altered in IBC *versus* non-IBC across three studies.
